# Breaking Bad News: A contextual model for Pakistan

**DOI:** 10.12669/pjms.346.15663

**Published:** 2018

**Authors:** Lubna Baig, Sana Tanzil, Syeda Kauser Ali, Shiraz Shaikh, Seemin Jamali, Mirwais Khan

**Affiliations:** 1 *Prof. Dr. Lubna Baig, MBBS, MPH, MMEd FCPS, PhD, APPNA Institute of Public Health, Jinnah Sind Medical University, Karachi, Pakistan*; 2 *Dr. Sana Tanzil, MBBS, FCPS, APPNA Institute of Public Health, Jinnah Sind Medical University, Karachi, Pakistan*; 3 *Dr. Syeda Kauser Ali MBBS, MHPE, PhD, Aga Khan University, Karachi, Pakistan*; 4 *Dr. Shiraz Shaikh, MBBS, FCPS, APPNA Institute of Public Health, Jinnah Sind Medical University, Karachi, Pakistan*; 5 *Dr. Seemin Jamali, MBBS, MPH, Jinnah Post Graduate Medical Centre, Karachi Pakistan*; 6 *Dr. Mirwais Khan, MBBS, MPH, International Committee of Red Cross, Islamabad, Pakistan*

**Keywords:** Breaking bad news, Communication, Health care providers, Violence

## Abstract

**Objectives:**

The purpose of the study was to identify the sequence of violence that ensues after breaking bad news and develop a contextual model of breaking bad news and develop a model contextual for Pakistan.

**Methods:**

A qualitative exploratory study was conducted using Six FGDs and 14 IDIs with healthcare providers working in the emergency and the obstetrics and gynecology departments of tertiary care hospitals of Karachi, Pakistan. Data was transcribed and analyzed to identify emerging themes and subthemes using thematic content analysis.

**Results:**

Impatience or lack of tolerance, lack of respect towards healthcare providers, unrealistic expectations from healthcare facility or healthcare staff were identified as main reasons that provoked violence after breaking bad news. A conceptual five step model was developed to guide communication of bad news by the health care providers. On initial testing the model was found to be effective in de-escalation of violence.

**Conclusion:**

Communication of bad news requires application of specific approaches to deal with contextual challenges for reducing violence against healthcare.

## INTRODUCTION

During past few decades Pakistan has seen an increase in the number of incidents of violence targeting the general population.^1^,^2^ The country has also observed a considerable increase in violence against healthcare mostly targeting healthcare providers (HCPs), ambulances and hospitals.^3^,^4^ There is a political will to effectively reduce these incidents not only against the healthcare providers but also the general public.

A multicenter study conducted in Karachi, in 2015 found that 66% of HCPs had experienced or witnessed at least one incidence of violence in last 12 months.^5^ The study also identified that verbal abuse was the most prevalent type of violence against healthcare providers. Relatively higher incidents have been reported from departments involved in provision of acute care particularly Emergency and Obstetrics and Gynaecology departments.^5^,^6^ Another multicenter cross-sectional survey of trainee physicians working in emergency departments of nine major tertiary care hospitals in Pakistan found that 76.9% of the physicians had experienced some kind of violence.^7^ The commonest reasons cited for increased violence on healthcare by patients’ attendants in Pakistan include; unreasonable expectations of patients and their families, poor quality of healthcare services and lack of trust on healthcare providers. Moreover, lack of education among general public and poor security measures in hospital and failure to communicate important information effectively to patients and their attendants has also been cited as reasons that incite violence on health care.^4^-^6^

Studies have identified that effective communication of health related information to patients and their families can reduce violence against healthcare.^7^-^9^ However, most of the people are reluctant to communicate any potentially disturbing or stressful information; considering it to be “Bad News”.^10^,^11^ Breaking bad news may lead to disappointment, distress and aggression in the receiver(s) and has a high potential to provoke violence depending on contextual factors.^10^,^11^

A qualitative study of experiences of healthcare providers regarding breaking bad news analyzed bad and good experiences separately and identified that interpersonal communication skills and specific training of healthcare providers regarding breaking bad news predominantly influenced outcomes of breaking bad news.^12^ The previous models of breaking bad news ignore the specific social context and have limited usability in countries like Pakistan.^11^,^13^ Hence, it is not only important to understand the reasons and context of violence against healthcare in response to breaking bad news but also the positive experiences of health care providers (HCPs) that helped mitigate those situations. This study was conducted to develop a contextual model for effective and safer communication of bad news in settings with high potential for violence against healthcare.

## METHODS

A qualitative exploratory study was conducted in August, 2016 in tertiary healthcare settings of Karachi to identify the reasons for violence related to breaking bad news and suggestions of participants in its management. Six Focus Group Discussions (FGDs N=45) and 14 in depth interviews (IDIs, N=14) were conducted with purposive sampling of HCPs working in the departments of Emergency, Obstetrics and Gynecology and Medicine of six public and private sector hospitals which included Jinnah Postgraduate Medical Centre, Civil Hospital, Ziauddin Hospital, Sindh Government Hospital-Korangi, Sindh Government Hospital-Lyari and Sindh Government Hospital-Malir. The respondents included; doctors, nurses, residents, dispensers and paramedics from seven public and private tertiary care hospitals. Using theoretical sampling the data collection was stopped when saturation was reached.

Semi structured interview guides were used after pre-testing. All the FGDs and IDIs were video-recorded and audio-taped. Data was transcribed and translated into “English” by a language expert. The transcripts of IDIs were discussed with the respondents to ensure the reliability and authenticity of the data.

Data was analyzed using thematic content analysis. Both ‘manifest content’ (visible, obvious components) and ‘latent content’ (underlying meaning) of the text were analyzed. The principal investigator (PI) and two co-investigators analyzed the data independently to identify codes and themes, which were finalized with consensus.

Ethical approval for the proposed study was obtained from Institutional Review Board (IRB) of Jinnah Sindh Medical University and relevant health care settings. Informed consent was taken from all study participants before the IDI or FGD.

## RESULTS

The study identified a number of reasons for violence against healthcare in response to breaking bad news. The causes of violence were classified as client and provider related causes. The client related causes included impatience or lack of tolerance, lack of respect towards healthcare providers, unrealistic expectations from healthcare facility or healthcare staff. A HCP from emergency department of a public sector hospital mentioned;

*“Violence occurs when people become emotional in response to adverse health outcomes “, (Emergency Medical Officer)*.

The provider-related causes included: bad attitude of HCPs towards patients and attendants, lack of timely communication, inability to counsel patients/attendants and appropriate preparation for undesirable outcomes. One study participant said;

*“Incompetency to counsel patients and their families and bad attitude of doctors with staff and attendants causes violence in healthcare settings”. (Nurse Supervisor)*.

The study participants suggested various strategies to help reduce violence in healthcare settings related to receiving bad news. Training in communication especially breaking bad news was suggested by most participants a postgraduate trainee said;

*“Training of health care providers in communication skills may help in reducing violence”*.

Most of the healthcare providers emphasized that violence in healthcare settings can be reduced by implementing specific patient-communication protocols. Based on the themes and subthemes emerging from data analysis a context specific training module was developed to train HCPs for effective and safer communication of sensitive information or breaking bad news. This model offers a contextualized stepwise approach to be used by healthcare providers for effective communication of bad news while ensuring healthcare providers` safety as well. Currently, this model is being implemented in some tertiary care teaching hospitals of Karachi and the effectiveness of the entire training on de-escalation of violence was found to be effective which also included the model on breaking bad news.^14^

## DISCUSSION

This is the first study which specifically explored ways to effectively manage breaking bad news in the local context. Our study identified lack of communication skills and empathic attitude among healthcare providers that resulted in violence against healthcare. This is in concurrence with the study done in Pakistan with residents related to breaking bad news.^15^ Previous studies have shown that empathic attitude of healthcare providers increases the effectiveness of management and also improves patient`s compliance to treatment and enhances their self-efficacy.^16^,^17^

**Box 1:**
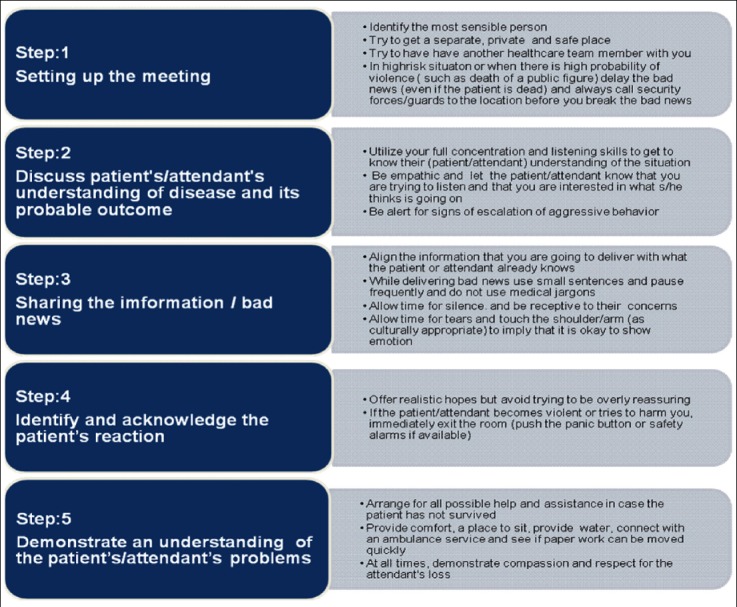
Details of breaking bad news model proposed to reduce violence in healthcare settings of Pakistan.

Our proposed model also emphasizes that training on communication skills is also important while implementing the step-wise approach for breaking bad news to patients and or their attendants. Our model has some similar characteristics/ constructs with a previous model Known as “SPIKE Strategy” for breaking bad news by Buckman. The SPIKE strategy focuses on demonstration of empathic attitude while recognizing and addressing emotions at receivers` end and is not specifically designed for communication of bad news.^18^

SPIKE does not offer essential guidance about identifying the right person for breaking the bad news in case many attendants are present. This in our opinion is very important in the context of Pakistan where families visit their patients in groups due to cultural practices and limited health literacy. Moreover, unstable political environment and extremism increases possibility to encounter political or religious mobs at hospitals and requires a specific strategy to deal with a larger group of emotionally heightened people.

A number of studies have identified the crucial role of cultural values and context for patient-centered communication in improving patient satisfaction and psychological well-being.^19^-^23^ Hence, our model also incorporates and applies cultural values by engaging attendants in prayers for recovery and life of a patient who arrived dead in case when they seem to be in denial or reluctant to hear any unwanted news.^19^ This can help to prepare them psychologically to hear bad news while diffusing anger and reduce chances of violence due to acute rage. This also provides sufficient time to the healthcare team to ensure necessary security arrangements as required by the situation.

Similarly, our model advocates using simple language and avoiding use of medical jargon while breaking the bad news to ease the uptake of information at receivers` end which is also supported by SPIKES.^18^ However, this approach is contrary to another model known as “P-A-T-I-E-N-T-E” Protocol developed specifically in the context of Brazil.^24^ This difference in the use of medical jargons or key terms for medical conditions may be explained on the basis of difference in the literacy rates of two countries. Hence, in Pakistan, where the literacy rate is very low the use of medical jargon or key terms while breaking bad news may cause confusion and may lead to violence.

Consequently, this study proposes first ever health communication model for Pakistan and provides evidence based guidelines to break bad news effectively in the context of Pakistan and similar cultures. Inclusion of study participants from healthcare settings frequently involved in breaking bad news from multiple hospitals of metropolitan city of Karachi increases trustworthiness of study findings. Analysis of data by more than two researchers establishes the credibility of the finding. Although the effectiveness of the model has not been tested independently but the effectiveness of de-escalation of violence training was found to be positive which also included training on breaking bad news model.^14^

### Disclaimer

The adaptation of this contextual model is dependent on basic communication skills of the healthcare providers; hence, this calls for basic trainings in communication along with adaptation of this contextual model. This model mainly addresses challenges of breaking bad news in emergency conditions in one episode and does not provide specific guidelines about role of frequent consultations to break the bad news if patient or attendants are not well prepared to receive the complete news or information as suggested by Dean and Willis.^19^

## CONCLUSION

Incorporating socio-cultural context in patient communication can improve their satisfaction and compliance to the information provided. The five step Model of Breaking bad news is a contextual model and can be tried in Pakistan and similar countries with local adaptation.
